# Autoimmune encephalitis with Anti-mGluR1 antibodies: a comprehensive review

**DOI:** 10.1007/s00415-025-13356-x

**Published:** 2025-09-11

**Authors:** Yun Chen, Lisha Xie, Hongmei Cui, Yali Zhang, Chaoer Wu, Wei Qian

**Affiliations:** Department of General Practice, The First People’s Hospital of Lin’an District, Hangzhou, Lin’an People’s Hospital Affiliated to Hangzhou Medical College, Hangzhou, 310000 Zhejiang Province China

**Keywords:** Metabotropic glutamate receptor type 1, Autoimmune encephalitis, Antibodies, Immunotherapy

## Abstract

Anti-mGluR1 encephalitis is a rare autoimmune disorder manifesting with cerebellar syndrome with varying levels of severity. However, limited data exist regarding the clinical features and treatment strategies for patients suffering from encephalitis associated with anti-mGluR1 antibodies. Herein, we comprehensively review and discuss clinical features of anti-mGluR1 encephalitis to enhance our understanding of this rare disorder. Our protocol was developed in accordance with PRISMA guidelines and is registered with the PROSPERO (identification: RD420251101607). To identify potentially relevant literature, we conducted a thorough search of the following bibliographic databases: PubMed, Web of Science, the Cochrane Central Register, and China National Knowledge Infrastructure. The search strategy yielded 402 articles, of which 23 met the inclusion criteria for our systematic review. These 23 articles, comprising 3 case series and 20 case reports, described 44 patients with anti-mGluR1 encephalitis. We independently extracted data on the following variables: publication, year, location, age, gender, associated malignancies, prodromal symptoms, clinical manifestations on the initial presentation, brain magnetic resonance imaging (MRI) findings, cerebrospinal fluid (CSF) testing, treatment, duration of last follow-up, and clinical outcome. From the current systematic review, cerebellar ataxia serves as the most prominent clinical manifestation in patients with anti-mGluR1 encephalitis. Furthermore, the proportion of patients receiving first-line immunotherapy was greater in the favorable prognosis group compared to the poor prognosis group. We underscore the importance of early immunotherapy to prevent irreversible cerebellar damage.

## Introduction

In recent years, the identification of a growing number of antibodies associated with encephalitis in the past few years had garnered significant clinical and scientific interest in the field of autoimmune encephalitis (AE) [1]. In 2000, researchers identified a novel antibody associated with AE: the anti-metabotropic glutamate receptor 1 (mGluR1) antibody, in two cases presenting with ataxia [2].

The mGluR1 is a G protein-coupled receptor prominently expressed in the dendrites of Purkinje cells within the cerebellar cortex, and also found in the olfactory bulb, thalamus neurons, globus pallidus, hippocampus, deep cerebellar nuclei, substantia nigra, and superior colliculus. These receptors play crucial roles in the development of cerebellum, synaptic transmission, plasticity, regulation, learning, memory, anxiety, and pain perception [3–6]. Autoantibodies targeting mGluR1 result in a rare form of autoimmune encephalitis, primarily manifesting as acute or subacute cerebellar ataxia. Diagnosis of this condition is confirmed by the detection of these antibodies in the serum or cerebrospinal fluid (CSF), along with clinical symptoms [7].

Stepwise escalation with immunotherapeutic approaches, such as intravenous immunoglobulin (IVIG), high-dose intravenous methylprednisolone (IV-MTP), and plasma exchange (PE), serves as the first-line immunotherapy for anti-mGluR1 encephalitis [8]. The mGluR1 antibody, functioning as an anti-cell surface antibody, facilitates neuronal dysfunction via humoral immunity and frequently demonstrates enhanced efficacy in immunotherapeutic applications. Nevertheless, the progression of the disease has been inadequately explored in the literature, with limited case reports available, and patient outcomes in anti-mGluR1 encephalitis remain variable and difficult to predict on individual basis. While some patients achieve a return to baseline function, others suffer from enduring severe cerebellar symptoms and long-term neurological complications. These outcomes are associated with the extent of neuronal damage present at the time of diagnosis, necessitating further investigation.

There is a scarcity of data on the clinical characteristics and therapeutic strategies for anti-mGluR1 encephalitis. This systematic review aims to consolidate and highlight existing research on anti-mGluR1 encephalitis to better understand this rare disorder.

## Methods

### Protocol and registration

The study followed the Preferred Reporting Items for Systematic Reviews and Meta-Analyses: (PRISMA) guideline. It has been registered with the PROSPERO International Prospective Register of Systematic Reviews (Registration number: CRD420251101607, accessible at: https://www.crd.york.ac.uk/PROSPERO/view/CRD420251101607).

### Data sources and search

We conducted an extensive systematic review by searching PubMed, Web of Science, the Cochrane Central Register, and the China National Knowledge Infrastructure from the inception of each database to July 2025. The search strategy employed both subject-specific and free-text terms, including “mGluR1”, “metabotropic glutamate receptor type 1”, “autoimmune encephalitis”, “autoimmunity”, “encephalitis”, “paraneoplastic”, “cerebellar”, “antibody”, “autoantibody”, and “autoantibodies”, combined with Boolean operators to ensure that all relevant studies are retrieved.

### Inclusion and exclusion

During the initial phase of the screening procedure, two independent researchers, Hongmei Cui and Yali Zhang independently reviewed the titles and abstracts of retrieved records to identify obvious exclusions. Articles deemed potentially eligible in this phase underwent a comprehensive full-text evaluation. Any disagreements encountered during the evaluation process were resolved through consensus. All articles that documented at least one case of anti-mGluR1 encephalitis were included, without language restrictions. Abstracts presented at letters to the editor, international or national conferences, and case reports or series were defined as eligible. The included patients met the diagnosis of anti-mGluR1 encephalitis was required according to the clinical findings of autoimmune encephalitis and the detection of antibodies against mGluR1 in the CSF or serum [9]. Patients were excluded if they met the following criterion: (i) Positive antibody testing for another neurological AE that more accurately explained their symptoms; (ii) Patients with insufficient information (i.e., lacking information on clinical presentation, treatment, and outcome) were excluded from the analysis.

### Data collection and extraction

Two reviews, Yun Chen and Yuner Hu, independently extracted the following data: patient number, publication details, year, location, age, gender, presence of prodromal symptoms, associated malignancies, clinical manifestations on the first presentation, CSF testing results, brain magnetic resonance imaging (MRI) findings, duration of the last follow-up in months, and clinical outcome. Any disagreements between the reviewers were addressed through further evaluation by a third reviewer. The collected data were entered into a spreadsheet accessible to the entire review team. All variables were standardized as follows: (i) Leukocytosis was defined as greater than > 5 cells/µl; (ii) elevated protein levels were defined as greater than > 0.45 g/L (with the conversion 100 mg/dL = 1 g/L); (iii) an increased IgG index was defined as greater than 0.7; (iv) Remission group (favorable outcomes) included patients with complete recovery or significant or mild improvement: (a) complete recovery: neurological symptoms are characterized by the complete disappearance of neurological symptoms, a return of neurological functions to pre-illness levels, and with no restrictions on dail1y activities; (b) significant recovery: a substantial improvement in neurological symptoms, with most neurological functions returning to pre-illness levels, but mild symptoms may persist or limitations on daily activities; (c) mild improvement: a partial improvement in neurological symptoms, but obvious functional disorders may persist and need help in daily life; no remission group (bad outcomes) included patients who exhibited stabilization, no improvement, or worsening of their condition. Missing information in the case was indicated as not available (NA)**.**

### Statistical analysis

Statistical analysis was conducted by SPSS version 26.0. Data following a normal distribution were presented as the mean and standard deviation, whereas data not following a normal distribution were shown as the median with the interquartile range (IQR). Categorical data were expressed as numbers and percentages. The chi-square test was used to estimate the relationships between selected variables and clinical outcome in patients. A P-value of less than 0.05 was considered to indicate statistical significance.

## Results

### Article included in search

**Fig. 1:**
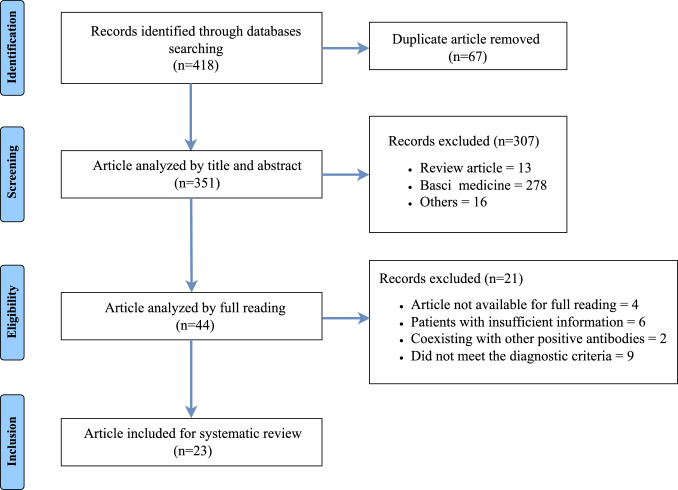
Flow diagram of selection process.

**Fig. 2:**
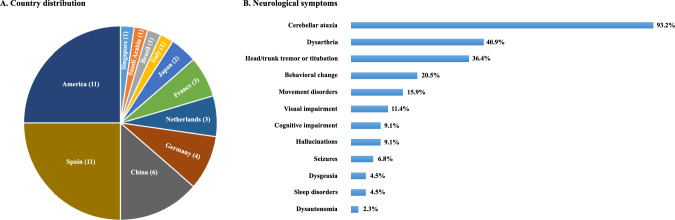
Clinical feature at presentation

See Fig. 1 for a flowchart of the search process. In total, 418 publications were identified in the first research. Following the exclusion of 67 duplicates, 351 articles were subjected to the first phase of screening. During the phase, 307 articles were excluded based on their titles and abstracts, including 13 review articles, 278 related to basic medicine, and 16 for other reasons. The remaining 44 articles underwent full-text analysis in the second screening phase, resulting in the exclusion of 21 articles due to inappropriate sample criteria. Specifically, 4 articles were not available for full reading, 6 involved patients with insufficient information, 2 coexisted with other positive antibodies, and 9 did not meet the diagnostic criteria. Consequently, 23 articles were deemed eligible and included in the qualitative analysis of the systematic review. These articles, published between 2000 and 2025, comprised 3 case series and 20 case reports, collectively documenting 44 patients with anti-mGluR1 encephalitis. The cases were geographically distributed as follows (Fig. 2): China (6), America (11), Spain (11), Germany (4), France (3), Netherlands (3), Japan (2), Italy (1), Brazil (1), Singapore (1), and Saudi Arabia (1).

### Clinical features

Overall, 44 patients with anti-mGluR1 encephalitis have been documented in our literature [2, 10–31]. Table 1 provides an overview of the demographic information and clinical characteristics of these cases, while Table 2 outlines the variables and their distribution throughout the progression of disease.

**Table 1:** Review of 44 cases with anti-mGluR1 encephalitis

No	References	Country	Age, Gender	Prodromal features	Main clinical features on first presentation	Associated malignancy	CSF analysis	Anti-mGluR1 Ab titers in serum/CSF	Brain MRI	Therapy	Months of follow-up time; Outcome
1	Song et al. [10]	China	7, M	Headache, fever 1w before	Gait instability, then developed dysarthria and limb ataxia	No	WBC: 286/µl, protein: 0.71 g/L, IgG: 92.8 mg/L, IgG index: 0.75, OCBs: pos	Serum: neg, CSF: 1:3.2	T1/T2/Flair hyperintensities in R cerebellum, DWI hyperintensities in cerebellar vermis	IVIG, IV-MTP, oral-Pred	1, significant improvement, MRI indicated decreased hyperintensities at 1 mo
2	Shen et al. [11]	China	56, M	Dizziness, nausea, vomiting 4 mo before	Gait instability, intention tremor, limb ataxia, and Spontaneous nystagmus	An abnormally increased metabolic modules in the thyroid (No biopsy)	WBC: 2/µl, protein: 0.56 g/L, OCBs: pos	Serum: 1:320, CSF: 1:100	Normal	IVIG, IV-MTP, oral-Pred, RTX, MMF	10, head tremor on initial therapy, then slow but significant improvement, can walk unassisted, serum CSF: 1:32 (10 mo)
3	Chen et al. [12]	China	50, M	Fever and dizziness 20d before	Slurred speech, unsteady gait, nystagmus, and limb ataxia	No	WBC: 190/µl, protein: 0.54 g/L, EBV: 4 sequences	Serum: 1:1,000, CSF: NA	Normal	IVIG, IV-MTP, MMF	36, partial improvement, still had cerebellar ataxia
4	Gui et al. [13]	China	38, M	Dizziness 2 mo before	Gait instability, limb ataxia	No	Protein: 0.485 g/L, Glu: 4.98 mmol/L, IgG: 0.689 mg/L, IgG index: 0.80, OCBs: NA	Serum: 1:100, CSF: 1:100	ischemic lesions in L basal ganglia	IV-MTP, oral-Pred, IVIG, RTX	6, complete remission
5	Khojah et al. [14]	Saudi Arabia	56, F	Fever 2d before	Slurred speech, unsteady gait, then developed double vision, head tremors, skew deviation, nystagmus, hypotonia, and limb and truncal ataxia	No	Lymphocytic: 6/µl, RBC:10 /µl, Glu: 6.5mmol/L, protein: 0.42 g/L, OCBs: pos	Serum: 1:1,000, CSF: 1:32	Bilateral, symmetrical, subcortical high signal intensity, mostly in the occipital lobes	IV-MTP, IVIG, RTX, AZA, oral-Pred, PE	60, mild dysarthria, bilateral dysmetria, and truncal ataxia, on maintenance dose and RTX every 6 mo,
6	Wang et al. [15]	China	7, F	No	Unsteady gait, swaying walking, easy falling, gait and limb ataxia	No	Normal, OCBs: neg	Serum 1:10, CSF: 1:10	Normal	IV-MTP, IVIG	3, complete recovery
7	Chandler et al. [16]	America	5, F	Nausea, vomiting, abdominal pain, fever, and headache within 24-h	Altered mental status with decreased speech, then unsteady gait	No	WBC: 240/µl, Glu: 3.6 mmol/L, protein: 0.77 g/L, OCBs: NA	Serum: NA, CSF: 1:64	T2 hyperintensity in the cerebellar vermis and adjacent cerebellar hemispheres, R > L	IV-MTP, IVIG	17, complete recovery
8	Vinke et al. [17]	Netherlands	50, F	Mild headache	Confusion, behavioral change with screaming and crying, following by stuttering, hallucinations, memory impairment, focal seizures	No	WBC: 6–10/µl, OCBs: pos	Serum: neg, CSF: pos	nonrecent vascular lesions, one in the vermis and the other in the R frontal cortex	IVIG, AZA, MMF	67, significant improvement of her well-being, with more energy and less memory problems, although experienced relapsing episodes
9	Goh et al. [18]	Singapore	15, M	NA	Progressive cerebellar dysfunction, ataxia gait, nystagmus, intention tremor, dysarthria, and dysdiadochokinesia	NA	Normal	Serum: pos, CSF: pos	Normal	Immunotherapy	3, significant improvement on cerebellar function
10	Sakashita et al. [19]	Japan	58, F	Dizziness 5 mo before	Broad-based gait, nystagmus, head titubation, truncal ataxia, and orthostatic dysautonomia	PET/CT: abnormal uptake in the uterus endometrium (no malignancy)	WBC: 1/µl, OCBs: neg	Serum: pos, CSF: NA	Normal	IV-MTP, IVIG	12, complete recovery
11	Liu et al. [20]	China	48, F	Fatigue 2w before, dizziness 1w before	Gait instability, limb ataxia, then dysarthria, choking cough while drinking water, nystagmus, sleep disorder, and hypotonia	NA	WBC: 29/µl, Glu: 3.4 mmol/L, Protein: 0.388g/L, OCBs: NA	Serum: 1:1,600, CSF: 1:1,600	Normal	IV-MTP, oral-Pred, IVIG	14, slow but significant improvement on dysarthria and nystagmus, still has limb ataxia, requires assistance for daily walking
12	Spatola et al. [21]	Spain	29, M	Weight loss, night sweats, fever	Difficulties falling asleep, then developed dysarthria, lower limb ataxia, behavioral changes (apathy, loss of initiative)	No	WBC:17/µl, OCBs: NA	Serum: + CSF: +	Normal	IV-MTP, IVIG, CYC, RTX	55, no improvement
13		Spain	22, F	Weight loss, headache, flu-like	Cerebellar syndrome, then developed hallucinations, memory deficits, and executive dysfunction	No	WBC: 214/µl, OCBs: pos, increased IgG index	Serum: + CSF: + +	Gadolinium enhancement of cerebellar leptomeninges	IVIG, RTX, CYC	12, Slow but significant improvement, can now sit and walk unassisted
14		Spain	45, F	Fatigue	Slowness in writing, hypophonia, dysarthria, then developed limb and gait ataxia	No	WBC: 3/µl, OCBs: neg	Serum: + CSF: + +	Normal	IVIG, IV-MTP, CYC, RTX, AZA	20, Significant improvement, still has mild dysarthria and limb ataxia, no gait ataxia
15		Spain	59, M	No	Slowly progressive gait and limb ataxia, dysarthria, wheelchair bound	No	WBC: 2/µl, OCBs: pos	Serum: + CSF: +	NA	No treatment	168, Stabilized over several years, then died of sepsis with multiple organ failure
16		Spain	54, M	Weight loss, fatigue	Cerebellar ataxia with repetitive falls, then behavioral changes with irritability, apathy, personality change, dysarthria, and bilateral visual loss	No	WBC: < 5/µl, OCBs: neg, increased IgG index	Serum: + CSF: NA	Subcortical dot-like resembling small vessel ischemic disease lesions in the frontal lobes	IV-MTP, oral-Pred, HQQ, MMF	120, cerebellar ataxia and vision loss reappeared when MFF and HQQ stopped for side effects, complete recovery after treatment was resumed
17		Spain	56, M	No	Behavioral changes with irritability, impulsivity, and apathy, memory problems, then vertigo, nystagmus, limb ataxia, gait instability, dysgeusia and seizures	No	WBC: 9/µl, protein: 0.66 g/L, OCBs: neg	Serum: + + CSF: + + +	Subcortical dot-like resembling small vessel ischemic disease lesions in cerebral hemispheres	IV-MTP, oral-Pred, MMF	90, mild improvement of irritability and gait instability
18		Spain	62, M	No	Gait instability, then developed leg myoclonia, then unable to walk	No	WBC: 27/µl, OCBs: NA	Serum: + CSF: NA	Old ischemic lesions in L frontal lobe	IV-MTP, oral-Pred	66, complete recovery
19		Spain	24, M	NA	Truncal and gait ataxia	Stage IIA HL	WBC: 4/µl, OCBs: NA Increased IgG index	Serum: + CSF: +	Normal	No treatment	6, stable with mild ataxia
20		Spain	38, F	No	Acute vertigo and gait unsteadiness, followed by diplopia, apathy, catatonia, then head titubation, neck and hand dystonia, dysarthria, severe gait, and limb ataxia, unable to walk, wheelchair bound	No	WBC: 2/µl, OCBs: NA	Serum: + + + CSF: + + +	Normal	IV-MTP, IVIG, RTX, CYC	45, mild improvement on dysarthria and limb ataxia, but severe gait ataxia persisted, wheelchair bound, although requires minimal assistance for everyday life activities
21		Spain	49, F	No	Focal seizures with impaired consciousness, then developed tremor, short-term memory loss, and behavioral changes	No	WBC: < 5/µl, OCBs: + , increased IgG index	Serum: + CSF: +	T2/FLAIR hyperintensities in cerebellar vermis and R frontal lobe	IVIG, AZA	84, complete recovery
22		Spain	6, M	Fever, headache, nausea	Rapidly progressive cerebellar syndrome, unable to sit unassisted, tremor and choreiform movements of face and fingers	No	WBC: 125/µl, OCBs: + increased IgG index	Serum: neg CSF: +	Normal	IVIG, IV-MTP	2.5, complete recovery
23	Bien et al. [22]	Germany	3, M	No	Unsteady gait with tendency to fall	No	Mononuclear: 28/µl, OCBs: pos	Serum: 1:20 CSF: 1:8	Normal	IV-MTP, oral-Pred	7, complete recovery
24	Chaumont et al. [23]	France	22, F	Coughing 1 mo before, temporal headache 5d before	Walking disability, and dysarthria, then developed gait, limbs, and truncal ataxia, dysarthria, hypotonia, areflexia, action tremor, nystagmus, oscillopsia, auditory hallucinations, and cognitive impairment	No	Lymphocytic: 214 × 10^3^ /µl, protein: 0.42 g/L, OCBs: pos	Serum: pos CSF: pos	cerebellar leptomeningeal contrast enhancement	IVIG, CYC, RTX	12, partial clinical improvement within 1 mo, with inconspicuous kinetic cerebellar ataxia at 12 mo
25	Gollion et al. [24]	France	64, M	No	Cerebellar ataxia, then could not walk unassisted, and developed myoclonic jerks	No	OCBs: pos (> 10)	Serum: NA, CSF: pos	Normal	IV-MTP, IVIG	4, significant improvement on ataxia, only very slight right-handed dysmetria
26	Christ et al. [25]	Germany	45, M	No	Progressive dysarthria	No	Lymphocytic: 7/µl, OCBs: neg	Serum: 1:1,000, CSF: 1:32	T2: discrete fluid-attenuated inversion recovery hyperintensity in the medial thalamus and L pulvinar	IV-MTP, oral-Pred, IVIG, RTX	24, significant improvement on dysarthria when RTX therapy, but with persistent mild gait ataxia, MRI showed a regression of the hyperintense lesion (6 mo)
27	Pedroso et al. [26]	Brazil	39, F	No	Behavioral change: apathy and catatonia, head titubation, then developed ataxia	NA	NA	Serum: 1:12,800, CSF: 1:512	Normal	NA	NA
28	Yoshikura et al. [27]	Japan	51, F	No	Dysarthria, dysphagia, and limb and truncal ataxia	No	WBC: 5/µl, protein: 0.29 g/L, Glu: 3.8 mmol/L, OCBs: NA	Serum: 1:3,200, CSF: 1:10	Normal	IV-MTP, oral-Pred, PE, TAC, AZA, IVIG, RTX	66, underwent repeated relapses on cerebellar ataxia after remission, required a walking frame to walk, serum anti-mGluR1 Ab: 1:100 (66 mo)
29	Lopez-Chiriboga et al. [28]	America	64, M	NA	Cerebellar ataxia, diplopia, nystagmus, and dysarthria	No	Protein: 0.43 g/L, OCBs: neg	Serum: 1:960, CSF: NA	Normal	Steroids, RTX	17, diplopia and nystagmus resolved; ataxia and dysarthria improved; relapsed when RTX discontinued
30		America	54, M	NA	Cerebellar ataxia	No	NA	Serum: 1:1,920, CSF: 1:256	NA	Steroids, IVIG	9, stabilized
31		America	81, M	NA	Cerebellar ataxia, cognitive impairment	No	Normal	Serum: 1:1,920, CSF: 1:64	Mild global atrophy, T2 hyperintensity in the central superior cerebellum	IVIG	24, ataxic speech and gait improved within 2 months; relapsed when IVIG discontinued and improved when resumed
32		America	77, M	NA	Cerebellar ataxia	No	Protein: 0.85 g/L, OCBs: neg	Serum: 1:61,440, CSF: NA	Cerebral atrophy	Steroids, IVIG	27, stabilized
33		America	51, M	NA	Paranoia, auditory hallucination, dysgeusia, followed by cerebellar ataxia	Remote history of testicular seminoma	WBC, 29/µl (90% lymphocytes), OCBs: neg	Serum: 1:7,680, CSF: NA	Normal	Steroids, IVIG, PE	11, Improved within 0.5 mo, from wheelchair to cane pendency
34		America	60, F	NA	Dysgeusia followed by cerebellar ataxia	No	Normal	Serum: 1:3,840, CSF: NA	Normal	Oral-Pred	168, improvement on dysarthria and gait ataxia
35		America	58, F	Herpes zoster 1 mo before	Diplopia, dysgeusia, vertigo, and ataxia	No	NA	Serum: 1:480, CSF: NA	None	None	6, improved spontaneously, but still persistent gait ataxia and dysgeusia
36		America	67, M	NA	Cerebellar ataxia	Cutaneous T-cell lymphoma	NA	Serum: 1:1,920, CSF: NA	NA	Chemotherapy for lymphoma	4, stabilized
37		America	67, F	NA	Bilateral hand paresthesia, vertigo, dysgeusia; then developed ataxia	No	NA	Serum: 1:960, CSF: NA	Mild cerebral and cerebellar atrophy	None	60, wheelchair-dependent
38		Germany	33, F	NA	Dysarthria, right hand ataxia, and cognitive impairment	Acute lymphocytic leukemia	OCBs: pos	Serum: 1:1,000, CSF: neg	T2 multiple brain and spinal cord lesions	Steroids	6, Improved, able to walk unassisted
39		Germany	77, F	NA	Ataxia, spastic paresis of the right leg, and cognitive impairment	Mantle cell NHL (bendamustine)	OCBs: pos	Serum: 1:3,200, CSF: NA	T2 multiple brain and spinal cord lesions	RTX	4, no change, required assistance to walk
40	Iorio et al. [29]	Italy	65, M	No	Unsteady gait, speech difficulties, then developed to dysarthria, limb ataxia, and nystagmus	Cutaneous T-cell lymphoma; Prostate adenocarcinoma	Normal protein and Glu, OCBs: NA	Serum: 1:200, CSF: 1:1	Normal except for a mild cerebellar atrophy	IVIG, oral-Pred	9, significant improvement on cerebellar ataxia
41	Lancaster et al. [30]	America	69, M	No	Ataxia, dysarthria, then nystagmus and difficulty in fixation of gaze	No	Cells: 8/µl, protein: normal	Serum: pos, CSF: pos	signs of small vessel ischemic disease	IV-MTP	NA, initially improved, stabilized for several years, and then died (cause of death unknown) 4.5 years after onset
42	Marignier et al. [31]	France	50, F	Diffuse transient headache	Gait disturbances, dysarthria, oscillopsia, head titubation, trunk and limb ataxia, and intense vertical nystagmus	No	Pleocytosis: 190/µl, protein: 0.72 g/L, IgG index: 0.60, OCB: normal	Serum: 1:20,000, CSF: 1:500	Flair/DWI diffuse abnormal hyperintensity in the whole cerebellum	IVIG, MMF, oral-Pred	40, significant improvement on cerebellar syndrome, serum anti-mGluR1 Ab:1: 1:500, MRI showed abnormalities decreased
43	Sillevis Smitt et al. ^[2]^	Netherlands	19, F	No	Truncal and gait ataxia, intention tremor	Stage IIA HL treated with chemotherapy (MOPP-ABV) and irradiation	Monocytes: 28/µl, protein, 0.28 g/L, IgG: 46 mg/L, IgG index: 1.2, OCBs: neg	Serum: pos, CSF: pos	Normal	oral-Pred, IVIG, PE	7, complete remission, serum anti-mGluR1 Ab: neg
44		Netherlands	49, F	No	Appendicular and truncal ataxia, dysarthria, and short-term memory loss	Stage II HL (nodular sclerosis type) treated with chemotherapy (BCVPP)	NA	Serum: pos, CSF: pos	Normal	PE	NA, no significant improvement on truncal ataxia, still requires assistance for daily walking

*Ab* antibody, *ALL* acute lymphocytic leukemia, *AZA* azathioprine, *BCVPP*: carmustine cyclophosphamide vinblastine procarbazine and prednisone, *CYC* cyclophosphamide, *EBV* Epstein-Barr virus, *F* female, Flair fluid-attenuated inversion recovery, *Glu*: glucose, *HL* Hodgkin's lymphoma, *HQQ* hydroxychloroquine, *IgG* immunoglobulin G, *IV* intravenous, *IVIG* IV immunoglobulins, *L* light, *M* male, *mGluR1* metabotropic glutamate receptor 1, *MMF* mycophenolate mofetil, *MOPP-ABV*: mechlorethamine vincristine procarbazine prednisone plus doxorubicin bleomycin and vinblastine, *MRI* magnetic resonance imaging, *MTP* methylprednisolone, mo month, *NA* not available, neg neg, NHL non-Hodgkin’s lymphoma, No. number, *OCBs* oligoclonal bands, pos pos, PE plasma exchange, *Pred* prednisolone, *R* right, *RBC* red blood cell, *RTX* rituximab, *TAC* Tacrolimus, *WBC* white blood cell

**Table 2:** Clinical features of 44 patients diagnosed with anti-mGluR1 encephalitis

Variables	Number of patients (n)	%
Total	44	
Age (year, IQR)	44	50.0 (30.0, 59.8)
< 18 (n, %)	6	6.5 (4.5, 9.0)
18–60 (n, %)	28	49.5 (38.0, 55.5)
> 60 (n, %)	10	67.0 (64.0, 77.0)
Gender	44	
Male (n, %)	22	50.0%
Female (n, %)	22	50.0%
Prodromal symptoms	32	
Yes (n, %)	17	53.1%
No (n, %)	15	46.9%
Associated malignancy	40	
Yes (n, %)	8	20.0%
No (n, %)	32	80.0%
Neurological symptoms	44	
Cerebellar ataxia (n, %)	41	93.2%
Dysarthria (n, %)	18	40.9%
Head/trunk tremor or titubation (n, %)	16	36.4%
Behavioral change (n, %)	9	20.5%
Cognitive impairment (n, %)	4	9.1%
Movement disorders (n, %)	7	15.9%
Sleep disorders (n, %)	2	4.5%
Seizures (n, %)	3	6.8%
Dysautonomia (n, %)	1	2.3%
Dysgeusia (n, %)	2	4.5%
Hallucinations (n, %)	4	9.1%
Visual impairment (n, %)	5	11.4%
CSF features	38	
Leukocytosis (n, %)	19	50.0%
Elevated protein (n, %)	8	21.1%
Increased IgG index (n, %)	9	23.7%
Positive OCBs (n, %)	12	31.6%
Anti-mGluR1 Ab		
Serum	42	
Pos (n, %)	39	92.9%
Neg (n, %)	3	7.1%
CSF	32	
Pos (n, %)	31	96.9%
Neg (n, %)	1	3.1%
Brain MRI	33	
Normal (n, %)	21	63.6%
Cerebellar atrophy (n, %)	3	9.9%
Cerebellar hyperintensity (n, %)	9	27.3%
Treatment	43	
Immunotherapy (n, %)	38	88.4%
First-line immunotherapy^a^ (n, %)	37	86.0%
Second-line immunotherapy^b^ (n, %)	19	44.2%
Combined First- and Second-line immunotherapy (n, %)	18	41.9%
Follow-up	43	
Months of follow-up time (mo, IQR)	41	14.0 (6.0, 57.5)
Clinical outcome		
Complete recovery (n, %)	9	20.9%
Significant or mild improvement (n, %)	25	58.1%
No improvement or Worsening (n, %)	9	20.9%

*Ab* antibody, *AZA* azathioprine, *CYC* cyclophosphamide, *IgG* immunoglobulin G, *IQR* interquartile range, IV immunoglobulins, *IVIG* IV immunoglobulins, mGluR1 metabotropic glutamate receptor 1, *MMF* mycophenolate mofetil, *MRI* magnetic resonance imaging, *MTP* methylprednisolone, *mo* month, *Neg* negative, *OCBs* oligoclonal bands, *PE* plasma exchange, *Pred* prednisolone, *Pos* positive, *RTX* rituximab, *TAC* Tacrolimus

^a^IV-MTP, IVIG, PE, oral-Pred. ^b^CYC, MMF, TAC, AZA, RTX

#### Demographic data

The median age at the first presentation was 50 years (IQR 30.0–59.8), with 22 patients (50%) being male. Patients under the age of 18 constituted 6 individuals (13.7%) of the total patient cohort, with a mean age of 6.5 years (IQR 4.5–9.0). Among the patients analyzed, 17 out of 32 (53.1%) exhibited one or more prodromal symptoms, which included fever, coughing, dizziness, nausea, vomiting, headache, fatigue, weight loss, and flu-like symptoms. Regarding associated malignancies, 8 out of 40 (20.0%) had a history of malignancy, of which 1 patient with cutaneous T-cell lymphoma and prostate adenocarcinoma occurring 36 months before and 18 months after the onset of encephalitis, respectively 5 patients with lymphoma; 1 patient with acute lymphocytic leukemia, and 1 patient with a remote history of testicular seminoma.

#### Neurological symptoms

Upon initial presentation, a significant majority of the patients (41 out of 44, 93.2%) experienced cerebellar ataxia (Fig. 2). Additional neurological symptoms were observed, including dysarthria (18 out of 44, 40.9%), head or trunk tremor or titubation (16 out of 44, 36.4%), behavioral change (9 out of 44, 20.5%), cognitive impairments (4 out of 44, 9.1%), movement disorders (7 out of 44, 15.9%), sleep disorders (2 out of 44, 4.5%), seizures (3 out of 44, 6.8%), dysautonomia (1 out of 44, 2.3%), dysgeusia (2 out of 44, 4.5%), hallucinations (4 out of 44, 91%), and visual impairment (5 out of 44, 11.4%). The behavioral changes encompassed paranoia, apathy, catatonia, irritability, and mood changes. Cognitive impairments were characterized by memory loss, executive dysfunction, and deficits in spatial orientation. Movement disorders included hypotonia, myoclonus, and choreoathetosis.

#### CSF analysis, anti-mGluR1 antibody, and brain MRI

CSF analysis showed increased leukocyte counts in 19 out of 38 patients (50.0%), elevated protein levels in 8 patients (21.1%), increased immunoglobulin G (IgG) in 9 patients (23.7%), and the presence of positive oligoclonal bands (OCBs) in 12 patients (31.6%). Antibodies against mGluR1 were identified in the CSF of 32 patients and in the serum of 42 patients. Specifically, antibodies against mGluR1 were identified in 39 out of 42 serum samples (92.9%) serum samples and 31 of 32 patients CSF samples (96.9%). In one patient, the antibodies were present in the serum but not in the CSF, while in three patients, they were tested in the CSF sample but not in the serum sample. Information on brain magnetic resonance imaging (MRI) at onset was available for 33 out of 44 patients. The brain MRI findings revealed cerebral atrophy, both non-enhancing and enhancing lesions in the brain and spinal cord, as well as cerebellar abnormalities, including cerebellar hyperintensity, enhancement of cerebellar leptomeninges, edema or atrophy. Among these patients, 21 patients (63.6%) exhibited normal MRI results, 3 (9.9%) had a cerebellar atrophy, and hyperintensity, 7 (21.2%) showed cerebellar leptomeningeal contrast enhancement, and 5 (15.2%) had ischemic lesions.

#### Treatment and outcome

In this study, 38 out of 43 patients (88.4%) were treated with immunotherapy. Among these, 37 patients (86.0%) were administered first-line immunotherapy, which included one or more of the following: IV-MTP, IVIG, PE, oral prednisolone (oral-Pred). In addition, 19 patients (44.2%) underwent second-line immunotherapy, which comprised cyclophosphamide (CYC), mycophenolate mofetil (MMF), tacrolimus (TAC), azathioprine (AZA), and rituximab (RTX). A total of 18 patients (41.9%) received a combination of both first- and second-line immunotherapy. At the last follow-up, with a median duration of 14 months (IQR, 6.0–57.5 months), 9 patients (20.9%) achieved complete recovery without any clinical sequelae, 25 patients exhibited significant or mild improvement, while 9 patients showed no improvement or experienced worsening of their condition. Two patients died. One patient initially showed improvement following six cycles of monthly corticosteroid treatment. It remained stable for several years, but ultimately died 4.5 years post-onset, with the cause of death remaining unknown. The other patient experienced progressive worsening of ataxia over several years and eventually died due to sepsis with multiple organ failure. Relapses were documented in 5 patients, consistently occurring upon discontinuation of immunotherapy. Symptoms re-emerged after cessation of treatment but improved or resolved upon resumption of immunotherapy. Notably, one patient required a walking frame to walk during follow-up.

### Correlated factors with clinical outcome

To explore the potential clinical factors associated with clinical outcomes, patients were categorized into a Remission group (n = 34) and a No Remission group (n = 9) based on their clinical outcomes (Table 3). Patients with poor outcomes were older, with a median age of 59.0 years compared to 49.0 years in those with favorable outcomes (p = 0.025). Additionally, the proportion of patients receiving first-line immunotherapy was greater in the favorable prognosis group (94.1%) compared to the poor prognosis group (55.6%) (p = 0.013). No significant differences were observed between the two groups regarding gender, prodromal symptoms, associated malignancy, neurological manifestations, CSF features, anti-mGluR1 antibodies, and MRI findings.

**Table 3:** Correlated factors with clinical outcome in patients with anti-mGluR1 encephalitis

Variables	Remission (34)	No remission (9)	P value
Age (year, IQR)	49.0 (22.0, 57.0)	59.0 (50.0, 72.0)	0.025
Male (n, %)	18/34 (52.9%)	5/9 (48.5%)	1.000
Prodromal symptoms (n, %)	16/26 (61.5%)	1/4 (25.0%)	0.290
Associated malignancy (n, %)	5/30 (16.7%)	3/9 (33.3%)	0.277
Neurological symptoms			
Cerebellar ataxia (n, %)	31/34 (91.2%)	9/9 (100.0%)	1.000
Dysarthria (n, %)	15/34 (44.1%)	3/9 (33.3%)	0.622
Head/trunk tremor or titubation (n, %)	14/34 (41.2%)	0/9 (0.0%)	0.019
Behavioral change (n, %)	7/34 (20.6%)	1/9 (11.1%)	0.622
Cognitive impairment (n, %)	6/34 (17.6%)	0/9 (0.0%)	0.312
Movement disorders (n, %)	6/34 (17.6%)	0/9 (0.0%)	0.312
CSF features			
Leukocytosis (n, %)	15/32 (46.9%)	3/5 (60.0%)	0.629
Elevated protein (n, %)	7/32 (21.9%)	1/5 (20.0%)	1.000
Increased IgG index (n, %)	9/32 (28.1%)	0/5 (0.0%)	0.207
Positive OCBs (n, %)	10/28 (35.7%)	2/3 (66.7%)	0.543
Anti-mGluR1 Ab			
Positive in serum (n, %)	28/31 (90.3%)	9/9 (100.0%)	1.000
Positive in CSF (n, %)	24/25 (96.0%)	5/5 (100.0%)	1.000
Both in Serum and CSF (n, %)	19/23 (79.2%)	5/5 (100.0%)	0.553
Brain MRI			
Atrophy (n, %)	2/33 (6.1%)	2/5 (40.0%)	0.076
Hyperintensity (n, %)	9/33 (27.3%)	0/5 (0.0%)	0.312
Treatment			
First-line immunotherapy (n, %)	32/34 (94.1%)	5/9 (55.6%)	0.013
Second-line immunotherapy (n, %)	16/33 (48.5%)	3/9 (33.3%0	0.477

*Ab* antibody, *CSF* cerebrospinal fluid, *IgG* immunoglobulin G, *IQR* interquartile range, *mGluR1* metabotropic glutamate receptor 1, *MRI* magnetic resonance imaging, *OCBs* oligoclonal bands

## Discussion

This study corroborates and extends the understanding of the clinical features associated with anti-mGluR1 encephalitis. Given the rarity of reported cases, diagnosing anti-mGluR1 encephalitis presents significant challenging. Our review of the literature reveals a predominance of cerebellar degeneration in all cases, including those involving individuals under the age of 18. The clinical manifestations in those younger patients did not markedly differ from those observed in adults. The mGluR1 receptor is highly present at the perisynaptic sites of Purkinje cell dendritic spines within the cerebellum [32]. In murine models, inhibition of this receptor causes cerebellar ataxia and difficulties in motor learning [33]. Mice deficient in the mGluR1 gene exhibit ataxic gait, intention tremor, and impairments in cerebellar long-term depression and motor learning [34]. Notably, dysgeusia was reported in 2 out of 44 cases. The localization of mGluR1 extends to the circumvallate and foliate papillae of the posterior mammalian tongue, regions implicated in umami taste perception [35]. Consistent with the presence of mGluR1 in the hippocampal formation, limbic symptoms such as psychiatric manifestations, memory loss, and seizures—occasionally co-occurred alongside ataxia. These findings suggest that antibodies targeting anti-mGluR1 can induce not only cerebellitis, but also a broader spectrum of neurological symptoms.

It is suggested that the degeneration of cerebellum related to antibodies against mGluR1 represents a ‘primary’ autoimmune disorder. Several cases (n = 8) have been identified as paraneoplastic. It could be hypothesized that the expression of mGluR1 by tumors acts as a trigger for an autoimmune response targeting mGluR1. Nonetheless, it is noteworthy that some cases exhibited a prolonged interval between the occurrence of the tumor and the onset of ataxia. In Hodgkin’s disease, for instance, the lymphoma precedes ataxia by several months to years, as observed in case 43 and 44 [2], with ataxia frequently manifesting during a prolonged complete remission. Smitt et al. did not detect mGluR1 expression in the tumor-containing lymph node of a patient with anti-mGluR1 encephalitis. Conversely, Iorio et al. demonstrated an abundant expression of mGluR1 in the luminal acinar epithelial cells of a patient’s prostate adenocarcinoma, along with binding of the patient’s IgG to mGluR1 [29]. Furthermore, aberrant expression of mGluR1 has been documented in other tumors, including breast cancer [36], melanoma [37], prostate cancer [38], and glioma [39]. In the context of anti-MGluR1 encephalitis, it is imperative to conduct thorough and comprehensive tumor screening. Our review indicated a strong association between anti-mGluR1 encephalitis and lymphoma. However, the initial symptoms of lymphoma are frequently atypical, and conventional imaging techniques such as CT and MRI may fail to detect occult lesions. Positron emission tomography/computed tomography (PET/CT), with its heightened sensitivity to metabolic abnormalities, aids clinicians in identifying sites of abnormally elevated metabolism within the body. The European Federation of Neurological Societies recommends that, in cases with a high suspicion of paraneoplastic syndrome, a negative CT scan should be followed by fluorodeoxyglucose-PET [40].

Currently, there are no established guidelines regarding the timing of treatment initiation for treatment of anti-mGluR1 encephalitis due to limitations in sample data. Although statistical analyses were not feasible, our experience with other autoimmune central nervous system disorders suggests that early immunotherapy may lead to neurological improvements. Continuous exposure of Purkinje cells to antibodies against mGluR1 can result in their degeneration, leading to progressive and irreversible cerebellar atrophy [41]. This is evidenced by observing patients who, despite their initial brain MRI appearing normal, develop cerebellar atrophy as the disease advances. Consequently, prompt and effective immunotherapeutic therapies are critical to avert irreversible injury to Purkinje cells. The treatment of anti-mGluR1 encephalitis involves immunosuppression, akin to the management of other AE. Overall, 9 out of 43 patients (20.9%) achieved complete recovery, while 25 (58.1%) experienced significant or mild recovery following immunotherapy. Although some patients experienced relapses after prematurely discontinuing, clinical symptoms improved or resolved upon resumption or treatment. The unsuccessful outcome of first-line immunotherapy necessitated the implementation of one or more subsequent second-line therapies; however, not all patients who received second-line treatment achieved complete remission, but remained stable for 12 months after therapy discontinuation. Notably, patient 28 underwent repeated relapses of cerebellar ataxia following remission, but remained stable for 12 months after receiving a regimen of RTX. Patient 26 maintained a stable clinical progression after initiating B-cell depleting therapy with RTX. Following two RTX cycles, CD19^+^ B cells were successfully and effectively depleted, constituting only 0.02% of the total lymphocytes. Patient 5 received a maintenance dose of RTX monthly and maintained a stable condition. RTX had demonstrated the ability to induce more prolonged and extensive B-cell depletion compared to other immunosuppressants, making it a viable option for second-line treatment.

Our study found that patients with poor outcomes were older at the peak of the disease course, and had a lower rate of receiving first-line immunotherapy compared to those with favorable outcomes. Spatola et al. [21] also observed that patients with poor outcomes exhibited greater neurological disability and were unable to walk unassisted at the peak of the disease, in contrast to those with better outcomes. However, no significant differences were identified between two groups regarding age, gender, clinical presentation, associated malignancy, CSF features, brain MRI findings, and anti-mGluR1 antibodies. It is important to note that the potential bias of data sources and the sample size may have contributed to the limited number of positive findings.

## Conclusions

We comprehensively review and analyze the clinical characteristics of anti-mGluR1 encephalitis to deepen our understanding of this rare disorder. From the current systematic review, cerebellar ataxia emerges as the most prominent clinical manifestation in individuals with anti-mGluR1 encephalitis. We emphasize the critical importance of early immunotherapy to avert irreversible cerebellar damage and prevent lasting sequelae.

## Limitations

Our study has some limitations, including the retrospective collection of clinical information, and the small sample size. The heterogeneity of the included case reports and case series, the potential influence of publication bias, and the variability in follow-up duration and reporting of treatment response. Language also represents a barrier that has impeded our ability to retrieve and access publications that were not written in English. We are unable to compare the differences between different immunosuppressive therapies in the future, only prospective, multicenter studies can address this question in the future.

## Data Availability

The data underlying this article will be shared upon reasonable request to the corresponding author.

## Abbreviations

AE: Autoimmune encephalitis
AZA: Azathioprine
CSF: Cerebrospinal fluid
CT: Computed tomography
CYC: Cyclophosphamide
IgG: Immunoglobulin G
IQR: Interquartile range
IV: Intravenous
IV-MTP: Intravenous immunoglobulins
IVIG IV: Immunoglobulins
mGluR1: Metabotropic glutamate receptor 1
MMF: Mycophenolate mofetil
MRI: Magnetic resonance imaging
NA: Not available
OCBs: Oligoclonal bands
PE: Plasma exchange
PET: Positron emission tomography
Pred: Prednisolone
PRISMA: Preferred Reporting Items for Systematic Reviews and Meta-Analyses
RTX: Rituximab
TAC: Tacrolimus
